# Antioxidant and Cytotoxic Effects and Identification of *Ophiocordyceps sinensis* Bioactive Proteins Using Shotgun Proteomic Analysis

**DOI:** 10.17113/ftb.59.02.21.7151

**Published:** 2021-06

**Authors:** Boon-Hong Kong, Chee-Sum Alvin Yap, Muhammad Fazril Mohamad Razif, Szu-Ting Ng, Chon-Seng Tan, Shin-Yee Fung

**Affiliations:** 1 Medicinal Mushroom Research Group, Department of Molecular Medicine, Faculty of Medicine, University of Malaya, 50603 Kuala Lumpur, Malaysia; 2 LiGNO Biotech Sdn. Bhd., Jalan Perindustrian Balakong Jaya 2/2, Taman Perindustrian Balakong Jaya 2, 43300 Balakong Jaya, Selangor, Malaysia; 3 Centre for Natural Products Research and Drug Discovery (CENAR), University of Malaya, 50603 Kuala Lumpur, Malaysia; 4 University of Malaya Centre for Proteomics Research (UMCPR), University of Malaya, 50603 Kuala Lumpur, Malaysia

**Keywords:** *Ophiocordyceps sinensis*, antioxidant activity, cytotoxic effect, bioactive proteins, protein-polysaccharide complexes

## Abstract

**Research background:**

*Ophiocordyceps sinensis,* a highly valued medicinal fungus, is close to extinction due to overexploitation. Successful cultivation of *O. sinensis* fruiting body (OCS02®) shows that the cultivar has a promising nutritional value and numerous bioactive compounds. Antioxidant and antiproliferative properties and biologically active proteins of the OCS02® are investigated for possible development into nutraceuticals.

**Experimental approach:**

The chemical composition of the OCS02® cold water extract was determined, and the antioxidant activities were examined using ferric reducing, DPPH^•^ and O_2_^•-^ scavenging assays. Tetrazolium dye (MTT) cytotoxic assay was performed to assess the antiproliferative activity of the extract. Bioactive proteins in the active fraction of the extract were identified using liquid chromatography (LC) and tandem-mass spectrometry (MS/MS).

**Results and conclusions:**

The OCS02® extract exhibited strong O_2_^•-^ scavenging (expressed as Trolox equivalents (18.4±1.1) mol/g) and potent cytotoxic activities against adenocarcinomic human alveolar basal epithelial (A549) cells (IC_50_=(58.2±6.8) µg/mL). High molecular mass polysaccharides, proteins and protein-polysaccharide complexes could have contributed to the antioxidant and cytotoxic selectivity of the OCS02®. LC-MS/MS analysis identified several potential cytotoxic proteases and an oxalate decarboxylase protein which may exhibit protection effects on kidneys.

**Novelty and scientific contributions:**

The findings demonstrate the potential of OCS02® to be developed into functional food due to its promising superoxide anion radical scavenging capacity, cytotoxic effect and presence of biopharmaceutically active proteins.

## INTRODUCTION

*Ophiocordyceps sinensis* or *Cordyceps sinensis* (in Chinese known as Dong Chong Xia Cao or 'worm in winter and grass in summer') is an insect-parasitizing fungus from the Ascomycetes family (*1*). *O. sinensis* is a traditional Tibetan, Chinese and Indian medicinal fungus found in Tibetan Plateau, China and Indian Himalaya (*2*). This fungus is commonly used as a functional food to reduce inflammation in the body, to improve respiratory system, libido and erectile function, and to treat liver, cardiovascular and chronic kidney diseases (*3*, *4*). It is also used as a type of herbal tonic to restore energy and promote general health (*3*, *5*).

Many scientific studies have shown that *O. sinensis* contains numerous bioactive compounds such as cordycepin, polysaccharides, sterol-type compounds, unsaturated fatty acids and peptides. These compounds exert various biopharmacological activities including anti-inflammatory, immunomodulatory, antiproliferative, anti-aging and antioxidant, as well as protective effects on the respiratory, hepatic, renal and cardiovascular systems (*6*). The use of *O. sinensis* as a medicinal health supplement is a global trend. However, natural production of this fungus is limited, and overexploitation to meet high market demand has led to near extinction of the species (*7*). Efforts in cultivation of *O. sinensis* using artificial media have been the most promising approach for mass production of *O. sinensis* for development into nutraceuticals. The artificially cultured fruiting bodies, mycelia and fermented mycelial products have been shown to possess biopharmaceutical properties comparable with the wild type, including antioxidant, anti-inflammatory, antitumour, immunomodulatory and anti-hyperglycaemic activities and the enhancement of neuromuscular activity (*8*-*11*).

Recent studies have demonstrated that a laboratory-cultured *O. sinensis* fruiting body (OCS02®) by LiGNO Biotech Sdn. Bhd. (Selangor, Malaysia) is safe for consumption. No toxic effects have been reported from an oral administration of 1000 mg/kg of OCS02® in rats in subacute toxicity assessment and no heavy metal was detected in the sample (*12*, *13*). It is rich in proteins and minerals, and contains high amounts of bioactive compounds including cordycepin, amino acids and glucans (*13*). Therefore, it is important to investigate the biopharmaceutical properties of OCS02® to support the development of this strain into functional food and nutraceutical. Previous study showed that the OCS02® cold aqueous extract possessed immunomodulatory properties attributed to its polysaccharide and protein contents (*14*). Herein, we aim to further examine the antioxidant and antiproliferative properties of OCS02® water extract, and to identify the potential bioactive proteins in it. The biopharmaceutical active proteins found in OCS02® could play a role as potential new drug candidates.

## MATERIALS AND METHODS

### Fruiting body OCS02® and extract preparation

The *Ophiocordyceps sinensis* fruiting body (OCS02®) was cultured using solid-state fermentation with rice-based medium as a substrate (LiGNO Biotech Sdn. Bhd., Selangor, Malaysia). This cultivated species was authenticated by its partial small subunit ribosomal gene (*12*). A mixture of 30 g freeze-dried OCS02® powder and 600 mL distilled water was stirred at 4 °C for 24 h to extract the heat-labile substances. The unextracted materials were pelleted using a refrigerated centrifuge (8000*×g*, 4 °C, 30 min; Sorvall Biofuge Primo R, Thermo Scientific, Waltham, MA, USA), while the water extract was filtered using a grade 1 filter paper (Whatman®, GE Healthcare Bio-Sciences AB, Uppsala, Sweden). The freeze-dried cold water extract was kept at -20 °C and dissolved in distilled water for further analysis.

### Fractionation of OCS02® cold water extract

The cold water extract of OCS02® was fractionated using gel filtration (Sephadex^TM^ G-50; GE Healthcare Life Sciences, Marlborough, MA, USA) column chromatography (*l*=40 cm, *d*=2.5 cm). The fractions were eluted using 0.05 M ammonium acetate buffer (Merck, Darmstadt, Germany). Fractions of three different molecular masses (low, medium and high) were collected according to protein and carbohydrate peak profiles. Bradford’s assay was performed to determine the protein content of the fractions (*15*). Carbohydrate content was estimated using phenol sulfuric acid assay (*16*).

### Isolation of proteins from the high molecular mass fraction

Proteins were precipitated from the high molecular mass (HMM) fraction using ammonium sulfate (Sigma-Aldrich, Merck, St Louis, MO, USA). The HMM fraction was dissolved in water, ammonium sulfate was gradually added until 100% saturation was reached, followed by continuous stirring for an hour at 4 °C. The precipitated proteins and non-protein component (supernatant) were retrieved by centrifugation and desalted using the Sartorius centrifugal concentrator, Vivaspin® 15R (Göttingen, Germany) of molecular mass cut-off value of 5 kDa.

### Total phenolic content

The phenolic content of OCS02® cold water extract and Sephadex-G50 fractions was determined using Folin-Ciocalteu assay (*17*). Briefly, Folin-Ciocalteu’s phenol reagent (Merck), 1:10 (500 μL), was mixed with a sample (10 μL) and incubated at ambient temperature (~22 °C) for 5 min. A volume of 350 μL sodium carbonate (115 µg/mL) was pipetted into the mixture and further incubated for 2 h. Gallic acid (Sigma-Aldrich, Merck) at concentrations from 20 to 200 µg/mL was used as standard. The absorbance values (765 nm) were recorded using a plate spectrophotometer (Bio-Rad model 680; Hercules, CA, USA).

### Antioxidant assays

Antioxidant activity of OCS02® cold water extract and its fractions was assessed using ferric reducing antioxidant power (FRAP) (*18*) and superoxide anion radical (O_2_^•-^) scavenging (*19*) assays. The 1,1-diphenyl-2-picrylhydrazyl radical (DPPH^•^) scavenging capacity was assessed using the method of Cos *et al.* (*20*), with slight adjustments. A volume of 25 μL sample (0-16 mg/mL) was mixed with 150 µL DPPH (Sigma-Aldrich, Merck) solution (40 µg/mL in methanolic solution). The sample was then incubated for 30 min in the dark (20-22 °C), and the absorbance was measured at 515 nm. Different concentrations (0-2 mg/mL) of Trolox (Sigma-Aldrich, Merck) were used to generate a standard curve.

### Cell culture and MTT cytotoxicity assay

American Type Culture Collection (ATCC^®^, Manassas, VA, USA) of human breast (MCF7 and MDA-MB-231), lung (A549) and prostate (PC3) adenocarcinoma cell lines, and human normal lung (NL20) cell line were used for this study. Roswell Park Memorial Institute (RPMI) 1640 medium (Nacalai Tesque, Kyoto, Japan) was used to culture MCF7, PC3 and A549 cell lines. MDA-MB-231 and NL20 cell lines were maintained in Dulbecco's modified Eagle's medium (DMEM) (Nacalai Tesque) and Ham’s F12 medium (Lonza, Basel, Switzerland), respectively. All the media contained 10% foetal bovine serum and cells were allowed to proliferate in an incubator at 37 °C with 95% humidity and 5% CO_2_.

To examine the cytotoxicity effect of OCS02® cold water extract and its fractions, cells seeded overnight (at optimal density) in 96-well microplate were treated with various concentrations (15.6-500 mg/mL) of samples (200 µL) for 72 h. After 72 h of treatment, MTT reagent was added into each well at a final concentration of 0.45 µg/mL and incubated for 4 h at 37 °C. The mixture of spent medium and MTT reagent was discarded, and dimethyl sulfoxide (DMSO) (200 µL) was used for dissolution of purple formazan crystals prior to measurement of the absorbance values (570 nm). Concentration of the extract and fractions that was required to inhibit 50% of cell proliferation (IC_50_) was calculated from the curves plotted using the cell viability percentage over the tested sample concentrations.

### Identification of proteins using LC-MS/MS

Proteins isolated from the HMM fraction were resolved with sodium dodecyl sulphate–polyacrylamide gel electrophoresis (SDS-PAGE) under reducing conditions. The separated protein bands were excised into 10 gel sections, where the gel sections were destained, reduced with dithiothreitol, alkylated with iodoacetamide and tryptic digested with trypsin protease (Thermo Scientific^TM^, Pierce™, Rockford, IL, USA) (*21*). Analysis was performed using an Agilent 1260 HPLC-Chip/MS Interface, coupled with Agilent 6550 Accurate-Mass Q-TOF LC/MS (Agilent Technologies, Santa Clara, CA, USA), following the protocol as described previously (*21*, *22*). The National Center for Biotechnology Information (NCBI) database of *Ophiocordycipitaceae* (non-redundant) was used for mass spectra searches that were performed using the Agilent Spectrum Mill MS Proteomics Workbench software packages. The spectrum mill settings applied including molecular ion (MH^+^) scan (100-3200 Da), complete carbamidomethylating of cysteines, peptides and protein scores greater than 6 and 20, respectively, scored peak intensity above 60%, and the significant number of distinct peptides is greater than or equal to two. Relative protein percentage was determined using the formula:

*w*(protein)=(*I*_m_/*I*_t_)∙*I*_r_∙100 /1/

where *I*_m_ and *I*_t_ are mean and total peptide spectral intensity of a protein and *I*_r_ is a relative intensity of each gel section in the protein lane estimated by densitometry using Thermo Scientific™ Pierce™ myImage Analysis™ Software, Rockford, IL, USA.

### Statistical analysis

All data are expressed as mean value±standard deviation (S.D.). Differences between the mean values in the experiment groups analysed using one-way analysis of variance (ANOVA) and Tukey’s HSD *post hoc* test (IBM SPSS Statistics v. 22) (*23*) were considered statistically significant at p<0.05.

## RESULTS AND DISCUSSION

### Antioxidant activity

Antioxidant activities including ferric reducing power, DPPH^•^ and O_2_^•-^ scavenging assay were performed on the *Ophiocordyceps sinensis* fruiting body (OCS02®) cold water extract and its fractions of different molecular masses (Table 1). The cold water extract demonstrated low FRAP and DPPH^•^ scavenging capacities compared to rutin and quercetin (positive controls). However, the capability of the OCS02® cold water extract to scavenge DPPH^•^ expressed as Trolox equivalents is ten times higher (0.015 mmol/g) than water extracts from the reported *O. sinensis* and other mushrooms (0.0013-0.0049 mmol/g) (*24*). The extract also demonstrated higher superoxide radical scavenger capability (18.4 mmol/g) than other reported *Lignosus* spp. mushrooms (9.61-9.90 mmol/g) (*25*, *26*). Three different molecular mass (HMM, MMM and LMM) fractions of the OCS02® cold water extract collected from Sephadex G-50 fractionation also demonstrated weak iron(III) reducing and DPPH^•^ scavenging activities, with the HMM fraction as the weakest DPPH^•^ scavenger. However, this fraction was the most potent O_2_^•-^ scavenger among the fractions with the activity higher than of the crude cold water extract and comparable to the positive controls. This superoxide scavenging property is of great significance as it implies that OCS02® can be beneficial as an antioxidant supplement to aid in prevention of superoxide anion radical-induced oxidative stress and related diseases. The antioxidant activity of the OCS02® was not correlated with its phenolic content. For instance, MMM fraction exhibited equal to or lower O_2_^•-^ scavenging activity than the cold water extract and HMM fraction, respectively, although it contains twice higher phenolic content (Table 1). A few studies have reported that the antioxidant activity of the *O. sinensis* is mostly contributed to polysaccharides (*27*, *28*). Thus, the strong O_2_^•-^ scavenging activity of OCS02® could be attributed to carbohydrates or polysaccharides that are abundantly present in the HMM fraction (Table 1). The synergistic effects among phenolics, proteins and protein-polysaccharide complexes could also have contributed to the antioxidant activities of the OCS02®.

**Table 1:** Chemical composition and antioxidant activity of the *Ophiocordyceps sinensis* fruiting body (OCS02®) cold water extract and its fractions

Sample	Chemical composition			Antioxidant activity		
*w*(protein)/%	*w*(carbohydrate)/%	*w*(phenolics as GAE)/(mg/g)	FRAP/(mmol/(min·g))	TEAC/(mmol/g)	
				DPPH	Superoxide anion
CWE	(2.1±0.3)^a^	(41.5±6.1)^a^	(6.7±0.6)^a^	(0.0022±0.0002)^a^	(0.0153±0.0001)^a^	(18.4±1.1)^a^
HMM	(3.8±0.9)^a^	(80.3±9.5)^b^	(5.2±1.7)^ab^	(0.0008±0.0002)^a^	(0.0027±0.0007)^b^	(22.6±1.9)^b^
MMM	(3.3±1.5)^a^	(28.0±5.1)^a^	(14.8±0.8)^c^	(0.0031±0.0003)^a^	(0.0141±0.0002)^a^	(15.5±0.2)^a^
LMM	n.d.	(0.6±0.3)^c^	(3.3±0.6)^b^	(0.0008±0.0002)^a^	(0.0170±0.0003)^a^	(2.6±0.5)^c^
Rutin	-	-	-	(2.6±0.11)^b^	(1.265±0.005)^c^	(29.1±1.3)^d^
Quercetin	-	-	-	(0.7±0.02)^c^	(1.214±0.003)^d^	(25.4±0.4)^b^

Protein and carbohydrate were measured on dry mass basis. All the values were expressed as mean±S.D. (*N*=3). Mean values in the same column with different letters in superscript are significantly different according to the analysis of variance and Tukey’s HSD *post hoc* test (p<0.05). Rutin and quercetin were used as positive controls in the antioxidant assay. CWE=cold water extract, HMM=high molecular mass, MMM=medium molecular mass, LMM=low molecular mass, n.d.=not detected, GAE=gallic acid equivalents, TEAC=Trolox equivalent antioxidant capacity

### Cytotoxic activity of OCS02® cold water extract and its fractions

**Fig. 1:**
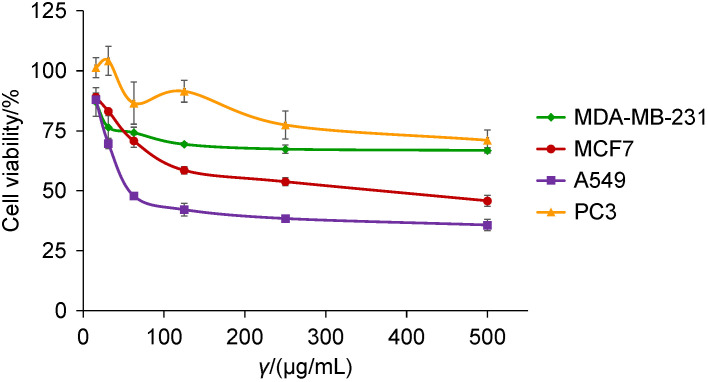
Cytotoxic activity of *Ophiocordyceps sinensis* fruiting body (OCS02®) cold water extract at various concentrations against MCF7, MDA-MB-231 (human breast adenocarcinoma), A549 (human lung adenocarcinoma) and PC3 (human prostate adenocarcinoma) cell lines. Values are expressed as mean±S.D. (*N*=3)

An investigation of the *in vitro* cytotoxicity of the OCS02® cold water extract showed that it exhibited significant cytotoxicity (IC_50_=(58.2±6.8) µg/mL) against lung cancer A549 cells (Fig. 1). The extract also exerted weak cytotoxic activity against MCF7 cells with the IC_50_=(371.0±62.0) µg/mL, or approx. 6-fold higher than against A549 cells. Our results showed that the cold water extract was more active in inhibiting the proliferation of oestrogen-dependent MCF7 breast cancer cells than the invasive, oestrogen-independent MDA-MB-231 breast cancer cells. There were no observed effects on the MDA-MB-231 and prostate cancer PC3 cells (IC_50_>1000 µg/mL). Although the cold water extract exerted good antiproliferative activity on A549 cells, it was cytotoxic to normal lung NL20 cells as well (IC_50_=(42.4±2.2) µg/mL). The NL20 is an immortalised non-tumourigenic lung cell line derived from human healthy lung epithelial cells through transfection with SV40 large T plasmid (*29*). NL20 cells showed no mutations in K-ras codons, no c-myc gene amplification and activation of dominant oncogenes (*30*) and are commonly used as non-tumourigenic (normal lung cells) lung cell model along with A549 lung adenocarcinoma cell model (*31*-*35*). With further fractionation of the cold water extract, the isolated HMM fraction demonstrated cytotoxic selectivity towards lung cancer A549 cells with selectivity index of 1.8 (Table 2). Yet, separated proteins and non-protein (mostly polysaccharides) components of the HMM fraction was cytotoxic to normal lung cell line (NL20), which implies the non-selective nature of the cytotoxicity of proteins and polysaccharides toward this cancer cell line. Previous reports have indicated that the polysaccharides from *O. sinensis* act on cancer cells by modulating the immune system rather than exerting direct cytotoxicity against the cancer cells (*36*, *37*). A recent work (*14*) done using OCS02® revealed that the HMM fraction consists of heteroglycans that stimulate the release of several cytokines/chemokines associated with its immunomodulator capability. Hence, this suggests that carbohydrates, the most abundant components in the HMM fraction, could act as immunomodulator associated with the antitumour effects on A549 cells.

**Table 2:** Cytotoxicity of *Ophiocordyceps sinensis* fruiting body (OCS02®) cold water extract fractions against human lung adenocarcinoma and normal cell lines

Sample	IC_50_/(µg/mL)		Selectivity index
A549	NL20
HMM	157.3±10.1	279.0±70.1	1.8
MMM	357.3±54.5	56.5±4.2	0.2
LMM	>1000	n.d.	n.a.
HMM-P	107.8±5.9	79.4±13.0	0.7
HMM-NP	213.3±37.5	95.3±14.1	0.5
*γ*(paclitaxel)/(ng/mL)	7.1±0.9	7.6±0.5	1.1

HMM=high molecular mass, MMM=medium molecular mass, LMM=low molecular mass, P=protein, NP=non-protein, A549=human lung adenocarcinoma, NL20=human normal lung, n.d.=not determined, n.a.=not available. Selectivity index was determined by dividing IC_50_ of NL20 normal lung cells with the IC_50_ of the A549 adenocarcinoma cells. Selectivity index above 1.0 revealed that the treatment was more cytotoxic (selective) against A549 adenocarcinoma cells

### Determination of the protein composition of HMM by LC-MS/MS

**Fig. 2:**
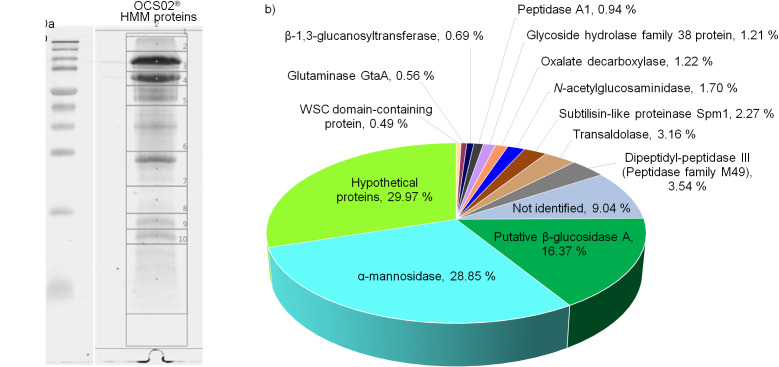
Protein profile of high molecular mass (HMM) fraction of *Ophiocordyceps sinensis* fruiting body (OCS02®) cold water extract: a) separation of the proteins on SDS-PAGE 15% gel, and b) distribution (in %) of the protein fraction of OCS02® identified by shotgun LC-MS/MS based on the NCBI non-redundant Ophiocordycipitaceae database

To date, limited studies are available for bioactive protein isolation from *O. sinensis* and their identification. Studies have shown that fungi contain potential antioxidative and cytotoxic proteins such as manganese superoxide dismutase, catalase, glutathione transferase, lectin, proteases and fungal immunomodulatory proteins (*38*-*40*). Using shotgun LC-MS/MS analysis, this study has identified a total of 17 distinct proteins in the HMM protein fraction (Table 3 and Fig. 2). Majority (>50%) of the proteins, *e.g.* α-mannosidase, β-glucosidase A, β-1,3-glucanosyltransferase, glycoside hydrolase family protein, transaldolase and WSC domain-containing protein, are involved in carbohydrate metabolism during the development of *O. sinensis* fruiting body. Study by Park *et al.* (*40*) demonstrated that a trypsin-like protease (CMP) purified from *Cordyceps militaris* has a significant inhibitory activity against human breast MCF7 and bladder 5637 cancer cells. We have identified several proteolytic enzymes including peptidase A1, peptidase family M49 proteins and subtilisin-like proteinase SPM1 in the HMM fraction. These proteases could have contributed to the cytotoxicity of the OCS02®.

**Table 3:** List of high molecular mass proteins from *Ophiocordyceps sinensis* fruiting body (OCS02®) cold water extract identified by LC-MS/MS

Gel section	*N*(spectrum)	*N*(distinct peptide)	Distinct summed MS/MS search score	Amino acid coverage/%	Protein pI	*I*_m_·10^5^	*w*(protein)/%	Database accession no.	Protein name
S1	2	2	31.80	2.1	6.26	1.23	0.65	799247974	Hypothetical protein HIM_04044
S1	2	2	30.12	2.2	5.91	5.10	2.70	908394288	α-mannosidase
S1	2	2	28.97	3.1	6.30	1.05	0.56	531866672	Glutaminase GtaA
S1	2	2	25.45	2.2	4.97	0.93	0.49	1032877594	WSC domain-containing protein
S2	2	2	37.54	2.8	5.63	3.28	13.07	1261512171	Hypothetical protein XA68_12018
S2	2	2	34.08	1.9	5.58	4.11	16.37	799246137	Putative β-glucosidase A
S3	3	2	37.75	3.1	5.91	1.46	17.86	908394288	α-mannosidase
S4	4	3	54.08	3.8	6.26	2.12	0.49	799247974	Hypothetical protein HIM_04044
S4	3	3	47.12	7.1	5.00	11.3	2.62	799246399	Hypothetical protein HIM_05392
S4	3	2	37.00	6.9	5.06	2.99	0.69	1008934073	β-1,3-glucanosyltransferase
S4	2	2	31.96	3.9	7.27	7.30	1.70	1008936229	*N*-acetylglucosaminidase
S4	2	2	28.87	3.0	5.39	2.51	0.58	1335267264	α-1,2-mannosidase
S4	2	2	28.30	2.3	4.46	21.6	5.01	908387070	Hypothetical protein TOPH_07589
S5	6	6	100.47	24.1	6.79	7.52	1.44	908389224	Transaldolase
S5	6	5	70.55	6.3	5.75	3.48	0.67	531863817	Peptidase M49, dipeptidyl-peptidase III
S5	5	4	69.02	4.6	5.91	6.02	1.15	908394288	α-mannosidase
S5	3	3	47.81	8.2	6.48	2.34	0.45	1261512568	Hypothetical protein XA68_11515
S5	3	2	35.49	5.9	6.20	2.36	0.45	799247347	Oxalate decarboxylase
S5	2	2	32.15	8.8	5.70	4.93	0.94	531867008	Peptidase A1
S5	2	2	28.71	4.2	8.94	6.10	1.17	799249484	Hypothetical protein HIM_02208
S5	2	2	28.45	2.3	4.46	26.9	5.13	908387070	Hypothetical protein TOPH_07589
S5	2	2	26.58	5.1	6.27	11.9	2.27	799247067	Subtilisin-like proteinase Spm1
S6	4	4	59.88	9.2	6.87	3.45	1.72	799247099	Transaldolase
S6	4	4	56.08	4.8	5.99	3.85	1.92	1339424435	α-mannosidase
S6	4	3	46.12	3.4	6.04	2.42	1.21	531865527	Glycoside hydrolase family 38 protein
S6	3	2	34.14	5.1	6.48	2.79	1.39	1261512568	Hypothetical protein XA68_11515
S6	2	2	33.76	5.9	6.2	1.55	0.77	799247347	Oxalate decarboxylase
S6	2	2	29.11	1.8	5.65	5.75	2.87	1335262293	Dipeptidyl peptidase 3
S7	2	2	22.19	1.3	6	5.80	1.21	1032877677	α-mannosidase
S8*	-	-	-	-	-	-	3.93	-	-
S9*	-	-	-	-	-	-	5.11	-	-
S10	3	3	42.77	2.8	5.91	3.87	3.43	908394288	α-mannosidase

* No protein was identified. *I*_m_=mean spectral intensity

*O. sinensis* water extract has been reported to have protective effects on kidneys including decreased proteinuria, enhanced renal functions and inhibited glomerular sclerosis (*4*). An oxalate decarboxylase (OXDC), an enzyme that mediates the degradation of oxalate, was identified in the HMM fraction of OCS02® cold water extract. Oxalate, a metabolic end product in humans, if present in excess, can cause calcium oxalate stones or kidney stones. A study has reported that oral administration of oxalate decarboxylase recombinant probiotic bacteria in hyperoxaluria rat models decreased the urinary oxalate level, thereby reducing hyperoxaluria (*41*). Several oxalate decarboxylase enzyme products such as ALLN-177 (clinicaltrials.gov/ct2/show/results/NCT02289755), Nephure™ (clinicaltrials.gov/ct2/show/NCT03661216) and Oxazyme (clinicaltrials.gov/ct2/show/results/NCT01127087) have undergone clinical trials and demonstrated promising results with significant reduction of oxalate levels in the OXDC-treated groups (*42*, *43*). The presence of oxalate decarboxylase in the HMM fraction implicates the potential use of OCS02® to improve renal functions.

## CONCLUSIONS

The extract from cultivated fruiting bodies of *Ophiocordyceps sinensis*, OCS02®, was shown to have promising antioxidant and cytotoxic activity with high content of polysaccharides, proteins and phenolics. The strong superoxide anion radical scavenging of OCS02® cold water extract is possibly mainly attributed to its high molecular mass polysaccharide content. The cold water extract inhibited proliferation of lung cancer A549 cells and oestrogen-dependent breast cancer MCF7 cells. The selective cytotoxicity of the high molecular mass (HMM) fraction against A549 cells is associated with the proteins and protein-polysaccharide complexes. Several bioactive proteins with potential cytotoxic properties and kidney protection effects including proteases and oxalate decarboxylase were found in the HMM fraction, implying that this fraction has the potential for development into dietary supplements as adjuvant therapy. Despite that, a more detailed study is required to gain better insights in the biopharmaceutical properties of HMM fraction. Our future study will focus on the investigation of the cytotoxic activity of this fraction *in vivo*, isolation of protein of interest and investigation of the biopharmaceutical properties and underlying molecular mechanisms of specific proteins for drug discovery.
